# Personalizing Cochlear Implant Care in Single-Sided Deafness: A Distinct Paradigm from Bilateral Hearing Loss

**DOI:** 10.3390/jpm15090439

**Published:** 2025-09-15

**Authors:** Emmeline Y. Lin, Stephanie M. Younan, Karen C. Barrett, Nicole T. Jiam

**Affiliations:** 1 Department of Head and Neck Surgery, University of California, San Francisco, CA 94143, USA; emmeline.lin@ucsf.edu (E.Y.L.); stephanie.younan@ucsf.edu (S.M.Y.); karen.barrett@ucsf.edu (K.C.B.); 2 Institute for Health and Aging, University of California, San Francisco, CA 94143, USA

**Keywords:** single-sided deafness, hearing loss, cochlear implant, precision medicine, music perception, big data, machine learning

## Abstract

**Background:** Cochlear implants (CIs) serve diverse populations with hearing loss, but patients with single-sided deafness (SSD) often show lower post-implantation usage and satisfaction than bilateral CI users. This disparity may stem from their normal contralateral ear providing sufficient auditory input for many daily situations, reducing the perceived need for consistent CI use. Consequently, uniform screening and evaluations, typically designed for bilateral hearing loss, often fail to address SSD’s unique needs. **Methods:** This narrative review synthesizes the current literature to explore patient and device factors shaping CI integration, outcomes, and experience in SSD. It highlights implications for developing personalized care strategies distinct from those used in bilateral hearing loss. **Results:** SSD patients face unique challenges: reliance on compensatory behaviors and significant auditory processing difficulties like acoustic–electric mismatch and place–pitch discrepancy. Anatomical factors and deafness of duration also impact outcomes. Traditional measures are often insufficient due to ceiling effects. Music perception offers a sensitive metric and rehabilitation tool, while big data and machine learning show promise for predicting outcomes and tailoring interventions. **Conclusions:** Optimizing CI care for SSD necessitates a personalized approach across candidacy, counseling, and rehabilitation. Tailored strategies, including individualized frequency mapping, adaptive auditory training, advanced outcome metrics like music perception, and leveraging big data for precise, data-driven predictions, are crucial for improving consistent CI usage and overall patient satisfaction.

## 1. Introduction

Since their first implantation over sixty years ago [1], cochlear implants (CI) have restored functional hearing to over a million individuals with severe to profound sensorineural hearing loss (SNHL), or hearing loss due to damage of the inner ear or auditory nerve, worldwide [2,3]. Originally intended for patients with bilateral SNHL, CI candidacy criteria in the United States were recently expanded in 2019 to include individuals with single-sided deafness (SSD) or asymmetric hearing loss [4]. SSD is defined as having normal or near-normal hearing in one ear but profound hearing loss in the contralateral ear, and affects approximately 0.11–0.14% of adults [5] and 0.2–0.5% of school-age children [4] in the U.S. While the normal-hearing ear often allows individuals with SSD to develop language and communicate more easily than those with bilateral deafness, the loss of binaural hearing and resulting asymmetry in auditory input still imposes significant challenges in daily listening situations [6,7]. These difficulties with sound localization and speech understanding in noise often lead SSD patients to seek treatment through hearing-assistive devices, such as a CI [7,8].

Like bilateral hearing loss, SSD may be either pre- or post-language acquisition, congenital or acquired, and present in individuals of any age. The most common etiology among all groups is sudden idiopathic SNHL [9,10]. Other causes of adult SSD include otitis media (chronic or cholesteatoma), cerebellopontine angle tumor and/or vestibular schwannoma, perilymphatic fistula, and head trauma [10]. Meanwhile, congenital or early-onset SSD can result from cochlear nerve deficiency, mumps and congenital cytomegalovirus infections, inner ear anomalies, and auditory neuropathy spectrum disorder [10]. There exist different etiologies for bilateral SNHL: among children, these include syndromic and non-syndromic hereditary or genetic disorders, congenital or postnatal infections, or complications from premature birth [11]. Among adults, reasons include presbycusis (age-related hearing loss), loud noise exposure, ototoxic drug use, and genetic mutations [12]. More details regarding etiology can be seen in Table 1.

Regardless of the etiology or duration deafness, patients with hearing loss face significant disability extending beyond difficulty with communication and affecting many dimensions of daily functioning. While these consequences are more profound among individuals with bilateral deafness, individuals with unilateral hearing loss are not exempt. Among adults, hearing loss may affect psychosocial well-being [20,21,22], occupational and professional opportunities [9], and increases the risk of comorbidities such as cognitive decline [23,24,25], ultimately impacting overall quality of life [26]. SSD impacts childhood development [27,28] too; children may demonstrate delayed language development, lower average IQ test scores, scholastic performance, and frequent misunderstandings in conversations [29,30]. Children may also be mislabeled as having attention deficit disorder or a learning disability and have higher school drop-out rates [31]. However, CIs have transformed this experience by restoring access to sound and speech, enabling individuals with hearing loss to more fully engage with the world around them. Studies have found that CIs improve language development and academic performance among children [32], improve peer and partner relationships [33,34,35], reduce rates of depression and anxiety [36], and significantly improve quality of life [37].

Despite these benefits, some patients discontinue CI use within a year or two after surgery, a phenomenon that is observed more frequently among patients with SSD compared to bilateral SNHL [38,39,40,41,42,43]. Given the vast heterogeneity among individuals who qualify for CIs, it is inevitable that their expectations and experiences with the device will likewise differ. Yet screening and candidacy considerations before surgery, as well as outcome measures after surgery, may not make adequate distinctions between unilateral and bilateral hearing loss conditions. Clinicians and their patients with SSD should acknowledge and understand the impact of retaining normal hearing in one ear on the perceived benefit of a CI for the other ear when determining the best course of treatment. The observed lower post-implantation usage [38,40,43] and satisfaction rates [39,40,42,44] of SSD individuals, compared to those of bilaterally implanted deaf individuals, therefore suggest the need for a more refined, personalized approach to care.

This narrative review summarizes the role of CIs in the clinical management of SSD in comparison to that of bilateral SNHL and offers potential recommendations for integrating a personalized medicine approach to the current strategies. Current perspectives and experiences among the SSD population that influence usage rates will also be discussed, as well as the utility of assessing music perception in both understanding the nuanced challenges and measuring outcomes among SSD patients. Additionally, this review explores how big data analytics can be further used to develop personalized treatment protocols, allowing for tailored interventions that optimize sensory restoration for both simple and complex sounds.

## 2. Methods

A thorough literature review was first conducted using PubMed, Web of Science, and Embase to source articles on the following topics: (1) current management strategies of SSD; (2) personalized medicine approaches to CIs; (3) CI usage rates in SSD; (4) CI difficulties and dissatisfaction among SSD; (5) music perception in CI users; and (6) applications for big data and machine learning algorithms. MeSH search terms included “cochlear implant”, “cochlear implantation”, “single-sided deafness”, “unilateral hearing loss”, “auditory perception”, “sound perception”, “music perception”, “music enjoyment”, “music experience”, “personalized medicine”, “precision medicine”, “big data”, and “machine learning”. Articles were reviewed by two authors (E.Y.L. and S.M.Y.) for their relevance to each topic, and their reference lists were further hand-searched to include any other relevant studies. Articles that were considered irrelevant, published in a language other than English, and/or consisted of only an abstract were excluded.

## 3. Current Clinical Management of SSD and Opportunities for Personalized Medicine

**Figure 1:**
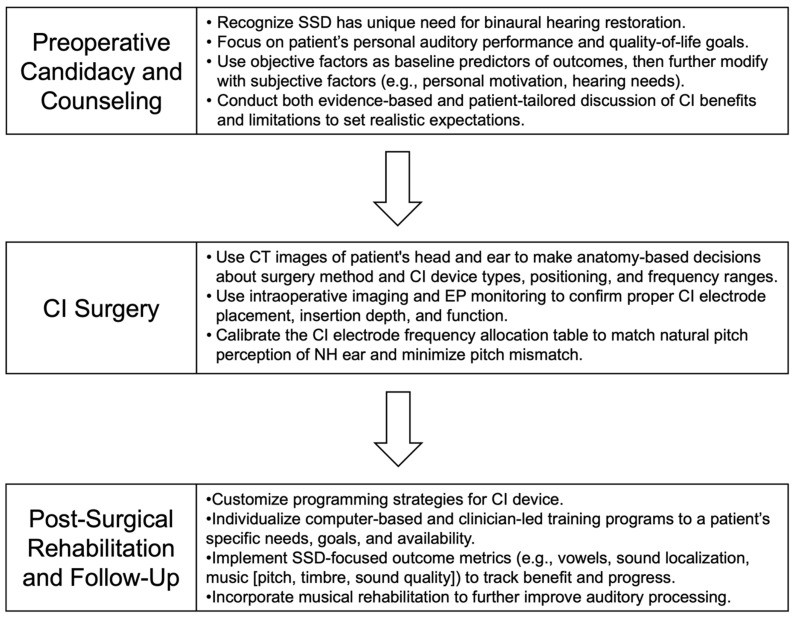
Cochlear Implants (CI) as treatment for single-sided deafness (SSD), and how a personalized medicine approach can be integrated to optimize the patient experience, maximizing CI use and minimizing dissatisfaction. CT = computed tomography. EP = electrophysiological. NH = normal hearing.

The clinical management of SSD is a complex, longitudinal process that often overlaps with that used for bilateral SNHL, despite important differences in patient needs and outcomes. In this section, we outline current practices and considerations for cochlear implant (CI) candidacy assessment, pre-operative counseling regarding the device and surgery, and strategies to optimize hearing outcomes following implantation in individuals with SSD. Given the distinct characteristics of the SSD population, we also explore how a multi-faceted, personalized medicine approach could be integrated at each stage of care to enhance patient-perceived benefit and satisfaction (Figure 1).

### 3.1. Treatment Options and Evaluation of CI Candidacy

Once diagnosed with irreversible SSD, patients may be offered one of three treatment options: a contralateral routing of signal hearing aid (CROS-HA), bone-anchored hearing aid (BAHA), or a CI. Though the most invasive of these devices, the CI is the only option that provides auditory input to the deafened ear, stimulating the auditory nerve with electrical impulses that the brain then interprets as sound. As a result, CIs may restore binaural hearing in patients with SSD to a greater degree than CROS-HAs and BAHAs, which only transmit the sounds from the affected ear to be heard and processed by the other ear [9]. While no devices currently enable patients with SSD to match the abilities of someone with two normal-hearing ears, the assistance that CIs provide for spatial hearing (often measured as ability to localize sound or hear speech in noise) can be a marked improvement compared to the un-implanted condition. CIs also have the added benefit of reducing tinnitus, which affects up to 80% of the SSD population and has a significant negative impact on quality of life [45].

CIs have only been approved for patients with single-sided deafness (SSD) in the past six years, following the FDA’s expansion of eligibility criteria to include individuals aged five and older with profound SNHL in the affected ear, defined as an average pure tone audiometry (PTA) hearing threshold of ≥80 dB HL [6]. Additional criteria include normal hearing in the contralateral ear (PTA ≤ 30 dB HL) and minimal to no benefit from a hearing aid in the deafened ear [46,47]. Other referrals for CI candidacy have followed the “60/60 guideline,” which includes an unaided PTA of ≥60 dB HL and a monosyllabic word recognition score of <60% in the affected ear. These thresholds have a sensitivity of approximately 96% and have expanded CI access worldwide [48]. While these values can help streamline determination of SSD candidacy, strictly following them can be double-edged. Individuals who experience significant functional challenges but do not meet the criteria are excluded from a beneficial intervention; meanwhile, those who qualify for, but will not benefit from, the CI unnecessarily undergo an invasive and costly procedure. Therefore, these guidelines are supplemented by thorough analyses of the patient’s performance on a variety of pre-operative audiological assessments, in the context of previously established outcomes data, to estimate the extent of auditory benefit a CI could provide.

In the United States, CI access for SSD is also determined—and limited—by the patient’s insurance provider. For example, Medicare currently does not cover CI for SSD, stating there is limited evidence for both the need and efficacy of this device in this population [49]. This policy may be adopted by other insurance companies as grounds to deny patients’ coverage; up to 34.5% of providers from a 2021 nationwide survey of CI clinics that provided at least 20 CIs per year reported that they had difficulty obtaining insurance coverage for their patients with SSD or asymmetric hearing loss. Additionally, the majority of survey respondents reported that SSD coverage was almost always denied on the first submission and required appeal via specialty external review to overturn the decision [49]. Some of these patients with SSD must wait for their other ear to deteriorate (if at all) to qualify for their first CI as a bilaterally deaf candidate; the consequences of this delay may include decreased rehabilitative potential of the CI [50]. Similar policies precluding SSD patients from receiving CIs exist in most countries with publicly funded healthcare systems worldwide. For example, the National Health Service in the United Kingdom [51], Medicare in Canada [52], and National Health Insurance in Korea [53] only cover cochlear implantations for individuals with bilateral SNHL but not SSD, even if the medical regulatory body has approved CI use for the latter population. One exception is Germany: under their Statutory Health Insurance, a unilateral CI is covered for patients with SSD given appropriate audiological evaluation and unsuccessful use of BAHA or CROS-HA devices [54], and was found to be a cost-effective policy for the nation [55].

Finally, when completing CI candidacy evaluations for unilateral and bilateral hearing loss, radiological examinations are key to confirming if the head and ear anatomy are suitable for implantation. Imaging is usually performed with magnetic resonance imaging (MRI) and/or temporal bone computed tomography (CT) and may help exclude patients with labyrinthine or cochlear ossification, inner ear malformations, or other anatomical lesions that may be important considerations for CI eligibility and counseling [6].

#### A Personalized Approach to Determining Candidacy

Although patients with SSD and bilateral SNHL often undergo similar clinical evaluations and candidacy assessments for the deafened ear(s), these approaches do not fully capture the distinct auditory challenges and goals associated with each condition. Following current recommendations relevant to bilateral SNHL when treating those with SSD carries two risks: excluding some SSD patients who would benefit from implantation while including others unlikely to use the device meaningfully, contributing to both a disappointing personal experience with the CI and a significant waste of national or institutional healthcare resources. Because the perceived severity of deficits associated with monaural hearing (i.e., hearing from one ear) can vary widely between individuals and listening environments, the evaluation of CI candidacy requires a multipronged approach. Quantitative audiometric thresholds and radiographic findings should serve as a baseline to be further supplemented or adjusted with a thorough understanding of patient-specific factors and experiences. Special consideration of the functional or quality-of-life consequences of SSD must be included. In children, this includes monitoring for limited progress in language or auditory development [56]; in adults, this includes considering communication deficits, trouble with balance or vertigo, and debilitating tinnitus [9]. Therefore, individualized counseling with the patient is a crucial next step when selecting interventions.

### 3.2. Pre-Operative Counseling with Patients

Pre-operative counseling is the process of providing patients with information, education, and support before a surgical procedure to ensure informed decision-making, reduce anxiety, and optimize perioperative outcomes. Through discussions with their hearing provider, patients are better able to understand whether the benefits of a procedure outweigh the risks.

The ability of unilateral CIs to improve sound localization and spatial speech recognition in noise has been well-established among SSD users [50,57,58,59,60,61,62]—a primary benefit that may help patients decide to proceed with surgery. Sound localization utilizes the functional binaural hearing possessed by individuals with two normal-hearing ears, as the different arrival times and intensities of the sound waves reaching each ear enable the brain to code and locate the source of the sound on a horizontal plane. These are known as the interaural time (ITD) and level (ILD) differences, respectively. Access to binaural cues is also fundamental for squelch and summation, both of which enhance an individual’s ability to hear speech in noisy conditions. Squelch is the brain’s ability to suppress background noise and increase the speech signal to improve understanding, while summation is the ability to combine similar auditory inputs from both ears to produce a stronger signal and enhance speech clarity [63]. CIs may provide some benefit for squelch and summation among both individuals with bilateral hearing loss or SSD, as a result of integrating input from both ears; however, the magnitude of this benefit and its clinical significance is variable in the literature [64,65,66,67]. This variability may be attributed to the CI’s limited sensitivity to fine timing differences [68], poor rate pitch discrimination [69], and impaired temporal fine structure processing [70]. Without these cues, CIs may only come close to, but not wholly restore, true binaural hearing.

Besides the potential improvements in spatial hearing measures mediated by central auditory processing, CIs also help with sound localization through the head shadow effect [64]. This refers to the attenuation of sound as it travels around the head to the far ear, and is helpful for understanding speech when it is spatially separated from noise (i.e., each arriving to a different ear), as the noise is naturally reduced to minimize any interference with the ear closer to the source of speech [62]. In SSD, this head shadow effect is lost when sound is presented to the deafened side, as it is all masked by the noise perceived by the normal-hearing side. However, with a CI in the affected ear, an individual with SSD may be able to detect the sound input, ultimately improving speech understanding in noise [64,71]. While the benefits in head shadow effect are more consistently described in the literature compared to squelch and summation, CIs overall help to provide some of the binaural advantages otherwise lost among individuals with SSD.

In reality, however, auditory outcomes and experiences with CI use may vary significantly between individuals [39]. Many factors contribute to this variability, which will be discussed in the next section. It is crucial, therefore, that pre-operative counseling includes thorough discussions between CI candidates and their healthcare providers to establish realistic expectations for post-surgical outcomes. Interviews with patients have highlighted a desire for more extensive counseling on how degraded sound quality can be through a CI, the amount of time needed for the brain to acclimate to the device, and how much effort and practice are needed to become comfortable with using the CI [72].

Furthermore, patients, especially those with acquired or post-lingual hearing loss, should understand that while CIs can significantly improve auditory function, they will not fully restore or cure their condition to their original state [73]. Similarly, special consideration of the normal-hearing ear must be made when counseling patients with SSD, who are able to compare the auditory performance of both ears in real time and therefore may develop unrealistic expectations for the level of sensory recovery of the compromised ear post-implant.

#### A Personalized Approach to Counseling

Pre-operative counseling provides an opportunity for developing a personalized model of care in SSD, as it considers both the patient’s unique objective and subjective factors [38]. Objective factors associated with functional outcomes include the patients’ medical history, such as the etiology and duration of deafness, inner ear anatomy, age at CI activation, and audiometric scores [6]. While these elements may not be modifiable, they can be used to predict the expected benefit of CI.

Subjective factors are equally important and can be evaluated using validated patient-reported outcome measures such as the Speech, Spatial, and Qualities of Hearing Scale (SSQ), Tinnitus Handicap Inventory (THI), and hearing loss-specific quality of life (QOL) instruments [6,74]. Counseling should explicitly address a CI candidate’s perceived impact of hearing loss on daily life, as well as explore their goals, daily listening needs, and expectations for device use and rehabilitation. Goals could include a desire for improved spatial hearing, tinnitus relief, or communication in complex settings, and a patient’s listening needs may depend on their auditory environments, frequency of social interactions, and lifestyle, all of which may differ substantially between individuals. Finally, as aforementioned, the patient’s previous experience with normal-hearing conditions impacts their expectations for their deafened ear [39]. Specifically, among the SSD population, where a “perfect” return to binaural hearing may be anticipated but is not achievable due to the mismatched auditory input, patients are more likely to be unsatisfied with their CI. These unmet expectations could contribute to patient dissatisfaction and non-use in the broader CI population [75,76].

The unique goals, needs, and expectations of the SSD population may further impact a patient’s subjective ability and motivation to comply with auditory rehabilitation strategies [9,73]. Personal commitment to consistent use and practice with the CI after surgery can be a limitation for progress, as the brain needs time, experience, and repeated exposure to adapt to a new modality of hearing. Patients must be clearly counseled that to obtain maximum benefit from their CI, they must be willing to keep it on even when it is uncomfortable or does not feel helpful [6]. For pediatric CI candidates, counseling is directed toward both the child and their family. This ensures that sufficient social and familial support is in place to promote consistent device use in a population that may face unique developmental and motivational challenges [77]. Family members play a critical role in encouraging daily device wear, facilitating auditory training at home, and creating supportive listening environments [56]. Additionally, preparing families for the gradual and possibly unpredictable nature of auditory development with a CI, especially when the patient has one normal-hearing ear to compare the implanted ear to, helps set realistic expectations and fosters long-term engagement with hearing rehabilitation [56].

Ultimately, it is essential to communicate the high degree of variability in hearing outcomes when an individual with SSD considers implantation. Hearing providers must help patients recognize how individualized factors such as anatomy, hearing loss etiology, consistency of device use, and auditory training post-implantation interplay to influence CI benefit. Setting realistic expectations through open, patient-centered discussions can help minimize the risk of post-implantation disappointment and device abandonment.

### 3.3. CI Surgery

Beyond choosing to proceed with CI surgery, a variety of other intraoperative decisions regarding the procedure and device must also be made between the patient and surgeon to promote the best possible outcome. There are many similarities in the surgical approach to and intraoperative decisions for CI surgery between patients with SSD and bilateral hearing loss, with each consideration potentially impacting post-operative outcomes. One example is selecting the design and length of the electrode. There are two predominant types of CI electrodes: lateral wall and perimodiolar [78]. Like their name implies, lateral wall (LW) electrode arrays are inserted against the outer, lateral wall of the scala tympani, one of three fluid-filled chambers of the cochlea. Their straight but flexible shape makes them useful among CI candidates with anatomical variations [79,80]. LW electrodes can range from 18 mm to 31 mm long, running almost the entire length of the basilar membrane [81]. It is thought that a longer electrode length allows for deeper insertion and broader cochlear coverage, including the apical regions (the innermost portion) of the cochlea, ultimately granting users greater access to spectral information of sound [82]. While some studies have demonstrated that a longer electrode may provide CI recipients with superior speech recognition performance both initially and long-term in comparison to shorter electrodes [83,84], other studies have concluded limited or contradicting results on the utility of a longer array and apical stimulation on auditory performance [81,85,86].

In contrast to the LW array, perimodiolar (PM) electrode arrays are shorter (16 to 25 mm) and pre-curved to hug the modiolus (inner wall of the scala tympani) more tightly, and thus may be able to stimulate spiral ganglion neurons with more focus and less electrical current [80]. This proximity may be associated with improved speech and melody perception scores during the first six months after operation, as compared to that of LW electrode users, but the differences seem to diminish after a year [87]. Ultimately, there may not be a significant difference in performance between the two types of electrode array designs [88]. Additionally, it was once thought that the fixed shape of the PM array was associated with an increased risk of translocation from the scala tympani into the scala vestibuli, which would damage the osseous spiral lamina and basilar membrane that separates the two compartments. Such trauma could cause ossification of the inner ear structures, negatively impacting both post-surgical auditory and vestibular outcomes [89]. However, more recent studies suggest that with correct initial placement and careful, atraumatic surgical technique, both PM and LW arrays yield similarly excellent outcomes and low overall complication rates [88,90,91].

Furthermore, the surgical technique, as well as the positioning and depth, of the electrode insertion may impact CI outcomes. Surgeons can access the inner ear and insert the CI electrode via the round window insertion (RWI) or a cochleostomy approach [92]. Considered less traumatic, associated with better residual hearing preservation, and lower likelihood of scalar translocation, RWI has increasingly become the procedure of choice [92,93,94]. Surgeons start with a posterior tympanotomy (incising and entering through the mastoid bone behind the ear) to open the facial recess (a small triangular cavity in the middle ear), allowing for natural access to the cochlea through the round window. The RWI technique is also associated with higher rates of successful electrode placement within the scala tympani [95], as these electrodes are positioned closer to the cochlear neural elements in the modiolus. This may improve both speech perception scores [95] and stimulus transmission efficiency [96], but also carries the caveat of damaging intracochlear structures if misplaced.

In comparison, a cochleostomy involves creating a new, separate opening in the cochlear wall, typically anterior and inferior to the round window. This technique allows the surgeon to customize the entry site and trajectory of the electrode, facilitating a more controlled, linear, and deeper insertion as the surgeon can align the electrode array closer to the centerline of the scala tympani [97,98]. However, cochleostomies also pose greater risk of inducing intracochlear damage. Thus, cochleostomy is reserved mostly for those cases where anatomical constraints preclude safe or easy round window access [95,99].

Given the integral role that a patient’s anatomy plays in CI surgical decision-making, surgeons need detailed pre-operative imaging to visualize the temporal bone, cochlear, and vestibular structures. Beyond determining a patient’s initial eligibility for surgery, CT scans are crucial for evaluating an individual cochlea’s size, width, and shape. Clear images from these scans enable more accurate measurements of the cochlea duct length and basal turn dimensions, which influence electrode selection, array length, and insertion technique [100]. Accurately calculating insertion depth and angle is critical for optimizing CI surgery safety and outcomes: if the electrode is over-inserted, this could also traumatize the cochlear duct, damaging neural structures nearby and any residual hearing [81]. If under-inserted, this may lead to insufficient coverage and therefore under-stimulation of the cochlea, leading to unsatisfactory functional outcomes [101].

Radiographic imaging can also be used intraoperatively to improve outcomes, especially for patients with difficult anatomy and/or when surgeons are uncertain about electrode placement [102]. Fluoroscopy and/or cone-beam CT provide real-time feedback that verify whether the electrodes were appropriately inserted within the cochlea, reaching the intended insertion depth and angle. These intraoperative imaging techniques may also detect whether any misplacement has occurred; examples include tip fold-over, incomplete insertion leading to extracochlear positioning of electrodes, or scalar translocation [103,104,105,106]. Additionally, optimal electrode placement can be confirmed via intraoperative electrophysiological monitoring. Post-insertion impedance telemetry (measuring the electrical resistance between the electrode surface and the surrounding cochlear tissues) provides objective information on both the proper location and functional integrity of each electrode contact [107]. Besides electrode mispositioning, abnormal impedance measurements may also indicate device malfunction or intracochlear trauma or bleeding, thereby enabling surgeons to make immediate corrections as needed to optimize surgical outcomes [108,109,110]. However, despite the well-documented utility of intraoperative imaging and monitoring to improve surgical precision, it is not a standard practice worldwide [111].

#### A Personalized Approach to Surgery

Ultimately, there is no singular, ideal electrode design or surgical technique that would confer maximum benefit to all CI users due to the unique anatomy and needs of each individual. Every surgical and device decision must be tailored to the patient’s clinical context through pre-operative discussions that present all the options alongside any available data on the outcomes associated with each; comparing results from prior candidates with similar clinical variables could especially be of help when predicting outcomes. This allows the patient to make a well-informed decision, highlighting the role of personalized medicine in this component of SSD treatment.

When choosing the electrode, special consideration must be made to address place-pitch mismatch, which is experienced by both implanted individuals with SSD and bilateral hearing loss with unilateral or bilateral CI. The place-pitch theory proposes that the perceived pitch of a sound is determined by the specific location (place) along the cochlea that is stimulated [112]. Thus, place-pitch mismatch occurs when the physical placement of the CI electrode contacts does not perfectly align with the tonotopic organization of the cochlea (the anatomic arrangement of auditory neurons based on their sensitivity to different frequencies of sound) [113]. Electrodes are typically positioned more basally in the cochlea than would provide ideal frequency-to-place mapping, as the arrays are limited in length and cannot reach the most apical (low-frequency) regions of the cochlea; surgical constraints further restrict insertion depth [114,115]. This results in inaccurate perception of pitches after electric stimulation.

In an attempt to reduce place-pitch mismatch, each electrode is assigned a specific frequency band calculated based on the average cochlea tonotopic map, but this Default Frequency (DF) approach is insufficient for patients with unique cochlear anatomy, as well as for patients with SSD [116,117,118,119]. Because SSD CI users can directly compare the pitch from their normal-hearing ear to the electrically evoked but inaccurate pitch from the CI, they may experience greater place-pitch discrepancy. Studies show that this population often experiences shifts in pitch up to two octaves apart, especially at low frequencies, and that this mismatch can negatively impact binaural fusion, speech intelligibility in noise, and sound localization [119,120,121,122].

A precision medicine approach via individualized frequency mapping can help address this issue to improve binaural perception and speech outcomes. One example is using anatomy-based fitting (ABF), which utilizes pre-operative imaging of an individual’s anatomical structures to precisely map both the angular insertion depth and position of each electrode contact within the cochlea. Then, the ABF approach tailors frequency band assignments to each electrode based on its actual tonotopic location, rather than using a generic or manufacturer-default frequency allocation table [123,124,125,126]. An additional strategy includes incorporating a subjective, interaural pitch-matching method to calibrate the frequency distributions of the CI to more closely match the tones heard by the contralateral normal ear. Creating these customized frequency allocation tables requires multiple testing sessions with the patient during the immediate post-CI activation period and therefore can be time-consuming. The benefits, however, are clear: implementing the modified frequency map improves speech intelligibility, sound localization, and sound quality among SSD CI-users compared to DF strategies [119].

### 3.4. Post-Surgical Auditory Rehabilitation

Post-surgical auditory rehabilitation, or any intervention that reduces the communicative and psychosocial consequences of hearing loss [127], is a key component of clinical management of this condition [73,128,129]. Ideally, these rehabilitation programs should begin immediately after activation and be administered routinely, in daily or weekly intervals [6]. However, there are currently no standardized aural rehabilitation methods in the United States for CI users with either SSD or bilateral SNHL [72]. One proposed model for CI recipients included four broad components: (1) sensory management, which is simply using the device to optimize auditory function; (2) instruction, regarding how to correctly use the device; (3) formal perceptual training, which uses stimuli to supplement learning opportunities beyond typical everyday communication to further improve patients’ auditory skills; and (4) counseling, to encourage participation in rehabilitation programs and teach patients how to emotionally and practically manage their hearing limitations [73].

Even within this model, there is great variability starting with sensory management through CI use. Fundamental to rehabilitation is the number of hours that patients use their processor each day. The association between device wear time and auditory performance has been well-studied among the adult and pediatric bilateral SNHL population [77,130,131,132,133,134,135]. Consistent CI use is important for both the immediate post-surgical period, when the recipient is first learning and adapting to listening via electrical input, and in the long term, allowing for maintenance of auditory progress. Clinicians typically recommend “full-time” device use, but the exact number of hours is subject to interpretation by both the patient and provider as there are no clear guidelines in place. For pediatric patients, one study found that wearing the CI for at least 80% of the age-appropriate “hearing hours,” or the number of hours the child is awake, is associated with better language outcomes [136]. Another study on adult patients recommended that using the CI processor for at least 10 h per day is needed to achieve the best speech recognition performance. They reported that average speech scores, namely for the consonant-nucleus-consonant (CNC) and Arizona Biomedical Institute sentence (AzBio) tests, increased by 3.0, 2.4, and 7.0 percentage points, respectively, with every hour of increased wear time after four weeks [132]. While average wear time among CI recipients is over 10 h per day [130,137], there is large intrapopulation variation within these studies, indicating that there are still many patients who may not be following these recommendations. For example, implanted SSD patients in particular may have CI lower usage and adherence rates because they are functionally less impaired when using their normal-hearing ear alone to listen, compared to when using their contralateral device [138].

Additionally, different perceptual training strategies exist, though their overall goals are similar: improve auditory awareness, discrimination, identification, and comprehension [139,140]. The wide heterogeneity in auditory rehabilitation protocols, stimuli, and efficacy described in the literature has contributed to inconsistent levels of implementation [72]. Limitations to the formation and use of well-defined training methods also include insufficient insurance coverage or reimbursement for these programs, and scarcity of speech-language pathologists to conduct sessions with patients [72]. As a result, the burden of post-surgical rehabilitation often falls upon the patients themselves, who are recommended to independently follow strategies that other CI-users self-report as beneficial. These include listening to audiobooks, watching movies with and without subtitles, engaging in computer-based auditory training (CBAT), or participating in CI support groups [72]. CBAT has been of particular interest as an intervention due to its time, resource, and cost accessibility for patients, but evidence of its utility in improving speech intelligibility and hearing ability is mixed [141,142]. Some studies reported improvements in sentence recognition in noise scores [143,144], consonant discrimination scores [128,143,145], and phoneme identification ability [146] within a few weeks of CBAT; others reported no improvement in sentence or vowel discrimination tests after a month of training [145]. Variability in regimen intensity and frequency, as well as participant adherence, are all factors that could contribute to the discrepancies in results. Thus, whenever possible, CBAT should be used as a supplement to, not replacement for, in-person auditory training, where patient engagement can be encouraged and verified [147].

Hearing provider-led auditory training programs have been piloted in small groups of implanted patients to varying degrees of success. For example, two studies that administered approximately ten one-hour-long training sessions to CI recipients reported minimal improvement in speech performance [148] but modest improvement in vowel recognition scores [149]. However, another short-term protocol featuring six weeks of auditory training and psychosocial counseling among 25 post-lingually deafened adult unilateral-CI recipients found clinically significant improvements on speech recognition outcomes compared to the control [150]. Besides the potential functional hearing improvements from increased practice and sound exposure, benefits of live auditory training include the opportunities to receive immediate feedback and motivation to persevere even when training is difficult [147].

#### A Personalized Approach to Rehabilitation

Multiple components of post-surgical auditory rehabilitation for individuals with SSD could benefit from a personalized approach to medicine. Virtual and in-person training paradigms should be carefully designed based on an individual’s auditory deficits, availability, and level of commitment. Moreover, these programs should be adaptive, evolving in response to the patient’s progress and changing needs.

One example of dynamic personalization involves real-time adjustments to CI technology throughout the rehabilitation process. During follow-up appointments, audiologists can modify CI electrode programming parameters—such as pulse width, stimulation rate, and signal amplitude—and assess whether these changes improve auditory performance [151]. Pulse width refers to the duration (measured in microseconds) of each electrical pulse delivered to the auditory nerve [152,153], while stimulation rate is the number of electrical pulses delivered per second to each electrode [154,155,156]. Signal amplitude is the strength of the electrical current delivered to each electrode and is the primary determinant of perceived loudness [6,157,158]. In addition, audiologists can leverage advanced CI features to fine-tune the sound processing mechanisms, helping patients with SSD achieve a more harmonious and comfortable integration between the CI and the normal-hearing ear [159]. Examples of these specialized programming considerations include directional microphone settings and loudness balancing. Adaptive directional microphones, which automatically focus on sounds from the front and attenuate noise from other directions, may better address SSD-specific auditory challenges compared to omnidirectional or “natural” microphone settings [6,160]. These challenges include the head shadow effect, spatial release from masking, and poor speech-in-noise performance [6,160]. Meanwhile, loudness balancing in SSD is typically achieved by adjusting CI stimulation levels so that both the implanted and normal-hearing ears perceive a stimulus at a comparable loudness [161]. While this approach may improve speech audibility in the absence of true binaural fusion, its effects on spatial hearing remain inconsistent, and some patients may prefer reduced CI intensity due to uncomfortable differences in sound quality between ears [6]. Ultimately, effective and personalized CI programming for SSD requires the integration of objective performance measures with ongoing, patient-centered feedback to optimize hearing outcomes and user satisfaction.

In addition, the CI’s sound processing algorithms can be adjusted to better align with the patient’s listening preferences. The most widely used sound coding strategy in the United States currently is Advanced Combination Encoder (ACE), which is the default for CI speech processors manufactured by the Cochlear brand. ACE works by dividing the incoming acoustic signal into multiple frequency channels (e.g., 22), then selecting a subset (e.g., 8–12) of channels with the highest amplitude (i.e., the most spectral information) for stimulation. By reducing the number of channels stimulated with each cycle, ACE limits channel interactions (i.e., when the signals from adjacent electrodes overlap and interfere with each other), thereby improving sound quality and speech perception [162]. In contrast, Continuous Interleaved Sampling (CIS), which was the primary coding algorithm in the past, stimulates all the channels in a rapid but interleaved pattern to minimize channel interaction [163]. In both cases, the temporal envelope of the incoming signal is processed while discarding fine temporal structure, thereby optimizing speech intelligibility at the cost of other abilities such as pitch perception and spatial hearing [157,164,165]. Alternatively, Fine Structure Processing (FSP) algorithms preserve elements of the signal’s fine temporal structure, potentially enhancing pitch perception, timbre recognition, and interaural timing cues—key aspects of binaural hearing and sound localization that are particularly relevant for individuals with SSD [166,167]. Several studies on CI users suggest that switching from CIS to FSP algorithms could improve speech and music perception test scores, as well as increase patient satisfaction with their device [166,168,169]. However, the effects of these coding strategies on SSD users remain unclear, as no studies to date have specifically evaluated this population. Therefore, investigating sound coding optimization for SSD represents an important area for future research.

Developing personalized outcome metrics to measure progress is another crucial component to auditory rehabilitation. A significant and confounding challenge in managing SSD CI users is the “ceiling effect” observed in standard clinical testing [170]. Traditional assessments such as speech-in-quiet tests (e.g., CNC words or AZBio sentence in quiet tests) are often insufficiently sensitive to capture the benefits and limitations of CI use among individuals with SSD. In fact, due to the high level of function provided by their normal-hearing ear, these patients frequently achieve maximum or near-maximum scores on these tests. As a result, these measures—when analyzed in isolation, without an experienced CI provider’s clinical judgment—may overlook the distinct challenges of distorted and disparate sound quality input that SSD patients face from hearing with two functionally different ears. This creates a profound clinical paradox: a patient’s chart may indicate an outstanding outcome, while the patient reports significant frustration with the device’s sound quality, a lack of perceived benefit in daily life, and a diminished overall quality of life [171]. The consequences of this disconnect are not trivial; it can lead to the dismissal of legitimate patient complaints and create difficulties in justifying insurance coverage for continued care, all the more highlighting the importance of clinicians using sensitive assessments to track subtle but meaningful changes in auditory processing over time.

To this point, the ceiling effect highlights the narrow scope of traditional tests, which fail to capture the complexity of real-world listening. Daily auditory life is not a series of words presented in a quiet room; it is a dynamic environment that demands the ability to follow conversations in loud restaurants, distinguish a specific voice among a crowd, and interpret emotional cues and inflections. Ultimately, it is in these noisy, spatially complex settings where patients with SSD face the greatest challenges to their hearing and not in ideal, quiet conditions. To address these issues, specific training to increase acclimatization to combined sound input, as well as testing for binaural integration skills, is necessary [172,173]. Examples include Bamford–Kowal–Bench sentence-in-noise (BKB-SIN) tests and adaptive hearing-in-noise tests (HINT) [9] with spatially separated target and masker for speech recognition, as well as sound localization tasks using multi-speaker configurations in a soundproof booth [6]. Additionally, by utilizing various noise configurations that mimic real-world listening scenarios, these programs and assessments demonstrate ecological validity and may be of greater functional benefit to these patients [173].

Furthermore, although the primary focus of CI rehabilitation remains the restoration of speech in both its received and produced forms, sound perception consists of many other complex dimensions that also warrant attention. Sound, especially that of music, contains both rich spectral and temporal information that current CI technology provides only limited access to [117]. Implementing outcome measures that assess fine auditory processing may more sensitively detect residual hearing deficits and help guide individualized rehabilitation goals. One such approach is to evaluate post-implant music perception ability or administer musical rehabilitation programs, both of which will be discussed in a later section. Ultimately, targeted training in complex sound processing has potential to discern auditory progress beyond what traditional speech-in-quiet tests reveal, offering deeper insights to improve overall outcomes in patients with SSD [118,174,175].

## 4. CI Usage Behaviors Among the SSD Population

Despite the well-documented benefits of CIs for individuals with SSD, some patients exhibit limited or non-use of their device, defined as wearing the CI less than the recommended number of hours per day or stopping use altogether, respectively [38,39,40,41,42,43]. Recent studies documenting this behavior are summarized in Table 2. These instances of limited daily wear time and long-term adherence highlight the importance of accurate candidacy assessments, patient-centered counseling, and individualized rehabilitation strategies when treating patients with SSD. These not only maximize the likelihood of successful, sustained device use, but proper counseling especially also prevents implanting individuals unlikely to benefit from this device from the very beginning.

To our knowledge, only one study has directly compared device wear time between patients with SSD and patients with bilateral hearing loss using either one (unilateral) or two (bilateral) CIs [38]. They found that across all ages, the SSD group actively used their CI for an average of 8.07 h per day. This was shorter than that of unilateral and bilateral CI users, who had an average wear time of 10.82 and 10.60 h per day, respectively. When results were further stratified by age, differences became significant between the 18- to 65-year-old (working age) group. Individuals in the working age cohort were also found to use their CIs less in quiet environments. It can be posited that in these circumstances, patients with SSD find their normal-hearing ear to be sufficient for listening and communication and therefore underuse their CI.

Deep et al. noted similar trends of limited use when analyzing the device datalogs of 47 SSD individuals, reporting a mean of 8.7 h used per day. Three of these subjects used their CI for less than 4 h per day, the main reason cited being listening discomfort [176]. Similarly, other analyses of device datalogs from patients with SSD revealed a mean daily wear time of 8 h [40] and 9.3 h [50]. While it appears that the SSD population wears their device on average one to two hours less than the overall CI population [38,130,137], it is unclear whether these differences have functionally significant effects.

In addition to decreased wear time, patients with SSD may stop using their CI completely, though the exact proportion of this population who do this varies. Elective non-use rates in SSD have been found to be between 4.4% to 14% [39,40,41,42], most within one year after activation. Some of the patients in these cohorts became unilaterally deaf at an early age and received the CI over ten years later, indicating how a prolonged duration of deafness may contribute to greater rehabilitation challenges and dissatisfaction [42]. Another smaller study of 8 individuals with SSD had a CI non-use rate of 62% (5) within four years post-surgery; in all of these studies, reasons discontinuation included lack of perceptible benefit in hearing and/or tinnitus [177].

Discontinuation of CI use may differ depending on the time after implantation. Tan et al. collected CI usage data among 54 and 38 patients with SSD at 12 and 24 months, respectively, post-implantation [44]. Not only did mean device usage time decrease from 8.2 to 7.0 h per day from the first to the second year, but the percentage of patients who became non-users nearly doubled from 9.3% to 18.4%, indicating that CI discontinuation may be a gradual process for patients [44]. Additional studies measuring elective non-use rates multiple years after implantation are needed to better understand the long-term behaviors, attitudes, and barriers among the SSD population. This data can then be compared to that of CI users with bilateral SNHL, whose non-use rates appear to be overall lower at 6.3% between 4 and 7 years after implantation and 11.0% at 7.5 years after implantation [185].

Usage patterns among pediatric SSD patients may vary considerably from those of adults, though both groups face challenges with consistent CI use. In a retrospective study of 66 children implanted for SSD, only 19% (10) were using their CI for at least 6 h per day, while 24% (13) reported usage between two to six hours per day. Notably, 18% (12) stopped attending audiology appointments and were considered lost to follow-up within two years post-surgery, contributing to the 52% (28) classified as non-users [43]. These findings aligned with the authors’ anecdotal clinical observations of low device use at their tertiary center. While the authors did not survey study participants on what drove these changes in behavior, some causes they speculated include limited family support and peer-to-peer interactions that discourage CI usage, such as bullying [43,186].

However, other studies have reported higher pediatric usage rates more closely resembling usage patterns seen in adults. For example, Ganek et al. reported average daily CI use of 6.22 h from the datalogs of 23 children with SSD [178], while Arras et al. reported 8.9 h from 12 pediatric SSD CI users [179]. In this latter cohort, 75% (8) were classified as “regular” users (≥8 h/day), while 25% (4) were limited users. Similarly, a meta-analysis by Benchetrit et al. including 119 children across 12 studies found that 74.3% (75) were regular users, 20.8% (21) were limited users, and 4.9% (5) were non-users [180].

Furthermore, like adult SSD patients, pediatric SSD patients demonstrate lower CI use compared to their bilateral SNHL counterparts. Mean wear time for implanted, bilaterally deaf children may be as high as 12 h per day, with the frequency of non-use at 6.8% and 12.3% at 5- and 10-years post-implantation, respectively [187]. When other follow-up time points were analyzed, non-use rates ranged between 0.96% to 3% of the sample population, while rates of limited CI use were 1.93% to 2% [188,189].

Ultimately, although the rates of limited or non-CI use among the entire SSD population are relatively low, these instances may represent a missed opportunity to achieve optimal auditory rehabilitation, especially since the procedure should have only been performed among individuals who were predicted to benefit through well-performed candidacy evaluations and pre-operative counseling. Beyond the personal cost to the patient, such as dissatisfactory sensory restoration and communication challenges, non-use also reflects a substantial financial burden on healthcare systems and payers, given the significant costs associated with CI devices, surgery, and post-operative care. Therefore, a more thorough understanding of the reasons driving these non-use behaviors is crucial for designing and implementing effective interventions that can increase CI usage rates among patients with SSD. Alternatively, understanding these reasons may also help clinicians identify the CI candidates who may perceive little-to-no benefit from the device in the real world, regardless of objectively positive performance on auditory assessments in the sound booth, and guide appropriate counseling away from surgery.

## 5. Factors Contributing to Lower CI Usage in SSD

**Figure 2:**
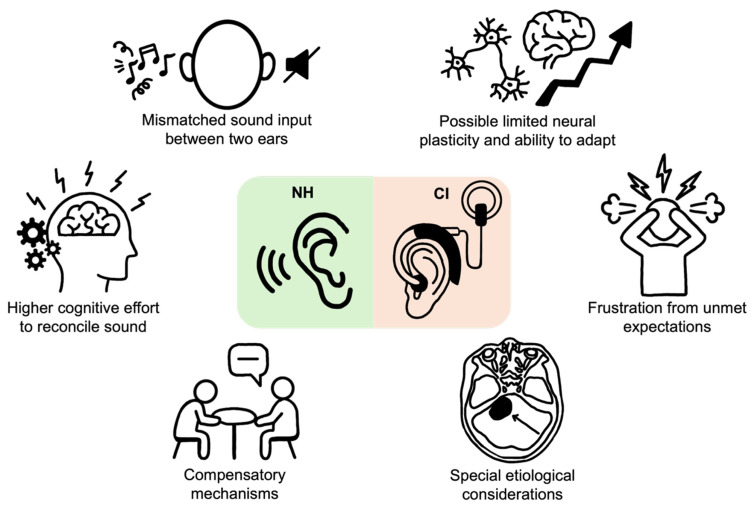
Factors contributing to reduced cochlear implant (CI) use and perceived benefit among individuals with SSD, recognizing that there is also significant intrapopulation variability in individual patient experiences and challenges. Special etiological considerations include duration of deafness, as well as contraindications such as vestibular schwannoma, and cochlear nerve deficiency. NH = normal hearing.

As mentioned earlier, CI usage rates, measured as either daily wear time or long-term adherence, are often lower than those observed in bilateral CI users. This disparity arises from a unique and complex set of challenges inherent to the SSD experience, which can often overshadow the device’s potential benefits (Figure 2). The constant presence of a normal-hearing contralateral ear creates a distinct auditory reality for SSD CI users, profoundly influencing their reliance on the device, their perception of its acoustic quality, their brain’s ability to process mismatched inputs, and their overall cost–benefit analysis of consistent use.

### 5.1. Reliance on the Contralateral Ear and Ingrained Compensatory Behaviors

A primary and intuitive reason for reduced CI usage in the SSD population is their continued reliance on the acoustic hearing of their contralateral, or “good,” ear. For many daily listening situations, particularly in quiet or one-on-one conversations, the hearing ear provides clear and sufficient auditory information, diminishing the perceived necessity and immediate value of the CI [190]. This functional adequacy of the normal-hearing ear often leads to situational or part-time use. Users may consciously choose to wear their device only in acoustically challenging environments, like noisy restaurants or large group settings, where the benefits of binaural hearing are most apparent [39]. In less demanding situations, the effort of wearing and processing sound through the implant may outweigh the subtle or negligible improvement over what the good ear can already provide.

Furthermore, individuals with long-standing SSD have typically spent years, even decades, developing and mastering highly effective compensatory strategies to navigate their hearing loss. These behaviors, like strategic body positioning to favor the hearing ear, actively turning the head to overcome the head-shadow effect, maintaining direct visual contact to aid in lip-reading, and requesting that conversational partners position themselves on the hearing side, become deeply ingrained and often subconscious [191]. While effective for communication, these adaptations can paradoxically reduce the perceived need for consistent CI use. The user has already established a functional, albeit unilateral, method of interacting with their auditory world. The CI, in this context, may be viewed as an accessory tool that requires active effort to use, rather than a primary and essential sensory input. This stands in stark contrast to bilaterally deaf individuals, who depend entirely on their implants for all auditory awareness and connection to the sound environment.

The concept of “listening effort” is also crucial. While the good ear provides sound, listening with only one ear, especially in noise, is cognitively taxing [192]. However, SSD individuals are accustomed to this familiar level of effort. The process of adapting to a CI introduces a *new* kind of cognitive load: the effort of learning to interpret an electronic signal and fusing it with the acoustic signal. For some users, the familiar, predictable effort of unilateral hearing is preferable to the novel and often frustrating effort of adapting to the CI, further contributing to intermittent use.

### 5.2. Auditory Processing and Binaural Integration Challenges

The ultimate goal of a CI for an SSD patient is to restore binaural hearing by enabling the brain to process and integrate sound from both ears. Achieving successful binaural integration, however, is arguably the greatest hurdle for this population and a significant source of user dissatisfaction. The brain is presented with a formidable task: fusing two fundamentally disparate types of auditory signals. The natural, acoustic hearing from the contralateral ear possesses rich spectral and temporal detail, while the electrical stimulation from the CI provides a more rudimentary, digitized representation of sound [193].

This acoustic-electric mismatch can be extremely difficult for the central auditory system to reconcile. A major issue is the stark difference in pitch perception between the two ears. Individuals with SSD experience significant place-pitch mismatch in their CI. This challenge is also well-documented in bimodal users, who must also integrate disparate acoustic and electric pitch percepts [170]; however, clinical observation and studies on neuroplasticity suggest that some of these users are able to adapt to this mismatch over time [174]. Nonetheless, for individuals with SSD, this acoustic-electric discrepancy can cause a single musical note or vocal tone to be perceived as having two different pitches simultaneously, leading to a distracting or unnatural listening experience [194].

In some cases, this mismatch can lead to a phenomenon known as binaural interference, where the input from the CI not only fails to help but actively disrupts or degrades the perception of sound compared to listening with the good ear alone [195]. The confusing signal from the CI can interfere with the clear signal from the normal ear, resulting in reduced overall speech clarity and a subjective sense of auditory chaos. These perceptions of suboptimal sound quality and distortion lead to listening discomfort and are a powerful deterrent to consistent CI use [159], as users may find it is simply easier and more effective to turn the device off.

Furthermore, the brain’s ability to adapt via neural plasticity is not infinite. In cases of congenital or long-standing pre-lingual SSD, the auditory cortex undergoes significant reorganization. The cortical regions normally devoted to the deaf ear are often re-purposed to process input from the hearing ear or even other senses, like vision [196]. This neural plasticity, while adaptive for unilateral hearing, makes it substantially harder for the adult brain to learn to process the new electrical input from the CI. The critical window for developing binaural pathways may have closed, limiting the potential for successful integration and capping the ultimate benefit of the implant [197].

These challenges are especially pronounced during complex auditory tasks. The brain’s ability to use the subtle ITD and ILD is crucial for localizing sound and suppressing background noise. The artificial nature of the CI signal can limit the precision of this processing, making these binaural advantages less robust than in individuals with two acoustic ears [62]. For some users, particularly those who rely on nuanced auditory cues for their profession or hobbies, this limitation can be a major source of frustration. For instance, musicians with SSD often report significant difficulties with pitch, timbre, and melody perception through their CI, as the device fails to transmit the harmonic complexity of music.

### 5.3. Anatomical, Pathological, and Device-Related Considerations

Finally, a range of anatomical, pathological, and device-related factors can physically limit the utility of a CI, leading to user dissatisfaction and reduced wear time. While these factors are relevant to all CI candidates, their impact is often magnified in the SSD population due to the constant comparison with the normal-hearing ear.

The underlying etiology of the SSD is a critical determinant of success. For example, patients with cochlear nerve deficiency, where the auditory nerve is congenitally small or absent, are poor candidates for implantation as there are insufficient neural fibers to receive the electrical stimulation from the device [198]. This is also a critical consideration for patients with a history of vestibular schwannoma, where the cochlear nerve may be compromised by the tumor or its surgical treatment, potentially impacting the overall success of implantation [199].

Other anatomical issues, while not exclusive to SSD, create a more pronounced perceptual conflict for these patients. For instance, while cochlear ossification—the formation of new bone within the cochlea, often occurring because of meningitis, temporal bone trauma, or labyrinthitis—can affect any CI candidate, the resulting need for a partial electrode insertion creates a limited, poorer quality sound signal. For an SSD patient, this distorted signal is constantly compared against the high-fidelity sound from their contralateral ear, heightening dissatisfaction and leading to device rejection [200]. The placement of the electrode array within the cochlea, known as the scalar location, is also vital. A suboptimal placement, such as translocation from the scala tympani to the scala vestibuli, results in less effective stimulation [201]. In the context of SSD, this does not just reduce performance but can actively worsen the overall auditory experience by creating a signal that is difficult for the brain to fuse with the clear acoustic input, potentially increasing binaural interference.

The duration of deafness is another profoundly important factor, with unique neuroplastic implications for SSD. Following the loss of hair cells, the spiral ganglion neurons—the cells that the CI directly stimulates—begin a slow, progressive process of degeneration [202]. More specific to long-standing SSD, however, is the significant reorganization of the central auditory pathways. The auditory cortex repurposes the deprived cortical regions, strengthening its reliance on the hearing ear [196]. This creates a formidable challenge for a new CI user: the brain must learn to interpret a novel electrical signal and integrate it with a highly efficient and dominant monaural pathway. This process of overcoming established cortical preference is a major hurdle to achieving binaural fusion and represents a distinct challenge not faced by traditional, bilaterally deaf CI candidates [197].

These anatomical limitations underscore the importance of thorough pre-operative imaging and counseling to ensure CI candidacy is appropriate and that patient expectations are aligned with potential outcomes. To further refine this personalized approach to SSD treatment, it is also necessary to explore more sensitive methods for assessing auditory processing and to leverage novel technologies that can help predict and enhance patient outcomes.

## 6. Special Considerations When Treating SSD

Addressing the behavioral, perceptual, and anatomical challenges discussed previously is fundamental to improving CI outcomes in the SSD population. However, to move toward a truly personalized model of care, clinicians and researchers must also look to emerging fields that offer more nuanced methods for assessment and more powerful tools for predicting success. This section will explore two such special considerations that represent the future of SSD management: the use of music perception as a sensitive probe for complex auditory processing and the application of big data and machine learning to tailor interventions and optimize patient care.

### 6.1. Music Perception as a Tool for SSD

Due to their normal-hearing ear, individuals with SSD often perform well on auditory tests that are difficult for individuals with bilateral SNHL, as these standard speech tests were not designed to measure or identify the specific kinds of challenges associated with unilateral hearing loss. This underscores a critical need for more sensitive outcome measures, which can probe the auditory system with a signal complex enough to reveal and quantify the real-world deficits that drive patient dissatisfaction and, ultimately, device non-use. One such example is musical perception.

#### 6.1.1. Music as a Uniquely Powerful Auditory Probe

Music is a unique, powerful candidate to fill this assessment gap. Its importance extends far beyond leisure; it is a fundamental and universal aspect of human experience, deeply intertwined with emotion, memory, social connection, cognitive health, and well-being [203,204]. For an SSD patient, the poor quality of music perceived through the implant can be particularly frustrating, as it often sounds distorted and unpleasant compared to the sound from their contralateral normal-hearing ear. In such cases, auditory input from the implant may be more distracting than beneficial, as an enjoyable musical experience typically requires accurate pitch representation and harmonic structure to convey emotion and meaning.

From a clinical and scientific standpoint, music’s value lies in its profound acoustic complexity. While speech is itself an acoustically rich signal, music often places distinct and broader demands on the auditory system. For example, music typically encompasses a wider frequency and dynamic range than conversational speech and is built upon precise pitch relationships that form melodies and the rich harmonic structure of multiple, simultaneous notes [205]. To be perceived successfully, music requires the auditory system to perform high-fidelity processing of this complex spectral and temporal information. Extensive research has shown that current CI technology, which is primarily engineered to transmit cues for speech, struggles to convey these fine-grained details [205,206]. Specifically, CIs are limited in their ability to transmit the fine temporal structure and provide the spectral resolution needed to separate harmonics, both of which are critical for an enjoyable musical experience. It is this mismatch between music’s acoustic demands and the CI’s capabilities that makes music an ideal ‘stress test’ for the auditory system.

#### 6.1.2. The Aural Mismatch: Deconstructing Music Perception Through a CI

When the complex signal of music is filtered through the technological and biological constraints of a CI, the acoustic signal is degraded, resulting in a profoundly altered and often frustrating perceptual experience. For most users, the rich, vibrant soundscape of music is reduced to a distorted, cacophonous, or robotic noise, stripping it of its emotional resonance [207]. This degradation stems from the fundamental limitations of delivering acoustic information via electrical stimulation, which is inherently poor at conveying the fine spectral details required for music. As a result, CI technology has been primarily engineered to optimize the transmission of cues for speech in quiet conditions, which are more robustly conveyed by the device [205].

The primary deficit lies in the perception of pitch. A CI’s limited electrode array cannot replicate the fine spectral resolution of a healthy cochlea, leading to a poor representation of place pitch. The logical solution of simply adding more electrodes to replicate the thousands of native hair cells runs into a fundamental physical barrier within the inner ear itself. Because the cochlea is filled with fluid, electrical current from any one electrode naturally spreads out, creating a “smearing” effect; if the electrodes were packed any closer, their electrical fields would blur into one another, making it impossible for the brain to perceive them as distinct pitches. Because of this limitation, some CI processing strategies attempt to convey additional pitch information through the rate of electrical pulses (temporal pitch), a mechanism that is insufficient for higher frequencies [206]. The limited spectral resolution makes it exceedingly difficult for users to discern melodies or appreciate harmony, with performance on recognition tasks often falling at or near chance levels [208].

A second major challenge is the perception of timbre. Timbre is defined by a sound’s harmonic structure, but CIs fail to transmit the rich upper harmonics that give each instrument its unique “color,” causing them to sound alike and creating a muddled, indistinct texture [209]. While rhythm perception is generally the best-preserved musical element due to the CI’s effective transmission of temporal envelope cues, it can even be compromised in complex passages [205]. For an SSD patient, this experience is particularly jarring, as their brain is forced to reconcile the rich, natural sound from their hearing ear with the distorted electric sound from the implanted ear in real time. This ongoing acoustic mismatch creates a constant state of auditory dissonance and is a major contributor to reduced device use and overall dissatisfaction, even among users with realistic expectations on CI outcomes.

#### 6.1.3. Clinical Applications: A Dual Role in Assessment and Rehabilitation

Given these inherent challenges, music perception serves a powerful dual purpose in the clinic. As an advanced outcome measurement, formal music processing tests can supplement traditional audiometry and provide a more complete picture of a patient’s auditory function. Standardized test batteries, such as the University of Canterbury Music and Auditory Perception (UC-MAP) skills test and the Clinical Assessment of Music Perception (CAMP), evaluate multiple domains, including pitch direction, melody, and timbre identification [210,211]. For the SSD user who performs at ceiling on speech tests (in bimodal listening condition), poor scores on a music test and musical sound quality assessments may objectively validate their subjective complaints, confirming that their dissatisfaction is rooted in genuine processing deficits and real-world bimodal listening conditions. This objective data is crucial for counseling, setting realistic goals, and guiding CI programming adjustments, such as modifying stimulation rate or frequency allocation to better match the user’s perceptual abilities. For example, if a music perception test reveals a patient has a pitch mismatch between the two ears, an audiologist can selectively adjust the frequency characteristic and boundaries for the corresponding electrodes to try to resolve bimodal perceptual listening conditions [122].

Beyond assessment, music is emerging as a powerful framework for auditory rehabilitation. The principle behind music-based training is that active, focused listening to a complex and challenging acoustic signal can drive neuroplasticity, enhancing the brain’s ability to interpret the impoverished signal from the CI [212]. A growing body of evidence demonstrates that this training leads to a transfer of effects, where improvements in music perception correlate with improvements in the critical domain of speech understanding in noise—a key challenge for all CI users [174,213,214]. Training programs often employ both analytic approaches (e.g., discriminating between two notes) and synthetic approaches (e.g., identifying a familiar melody). These programs, many now available as accessible computer-based software, guide users through engaging tasks that challenge their auditory system in a structured way [174]. For the SSD population, this therapeutic approach is particularly promising. It is inherently more engaging than rote drills and provides a structured method for encouraging use of the implanted ear, with the goal of enhancing the subjective benefit of the CI and fostering more consistent, long-term use.

### 6.2. The Role of Big Data Within Personalized Medicine for SSD Management

Big data, which refers to the large and complex datasets that can be mined for patterns and knowledge [215], is another tool that can be utilized to inform personalized clinical management of SSD. Because of the overall low rates of CI use in the SSD population, most of the published studies regarding SSD CIs have been conducted with small samples and therefore have low statistical power. Additionally, most existing datasets are represented by traditional CI candidates and do not reflect the expanding CI candidacy changes and SSD users. This may lead to results that are nongeneralizable to SSD patients [216,217]. The big data approach offers a solution to this limitation. By combining SSD data points across institutions and employing federated learning techniques, analyses using big data contain sufficient statistical power to identify true or previously hidden relationships between factors that affect SSD CI candidacy considerations, usage rates, and auditory outcomes [218,219]. These associations can be used to support nuanced, evidence-based guidelines and recommendations for individuals with SSD, allowing patients and providers to choose the CI intervention with more confidence. This information can also be used to identify and screen out patients who may not benefit from a CI, increasing the chances that those who ultimately undergo the procedure have the clinical factors, personal expectations, and rehabilitative resources to optimize outcomes and consistent device use.

To truly advance personalized care for this population, a critical next step is the establishment of a large-scale, multi-center or even nationwide clinical data registry specifically for SSD CI recipients, after first ensuring that institutional CI databases had representation of SSD CI users. Such a database, which is a clear application of big data in healthcare, would pool de-identified patient information from numerous clinics, creating a powerful resource for research [220]. Initiatives like the Auditory Implant Initiative [220] and the American Academy of Otolaryngology’s Regent registry [221] are already moving in this direction by creating repositories that include patient demographics, hearing history, etiology of hearing loss, surgical details, and longitudinal performance data. By aggregating this information, clinicians and researchers can mine the data to overcome the limitations of single-center studies and identify robust patterns in the SSD population that were previously undetectable.

Big data is also essential for training effective machine learning (ML) algorithms, which can be clinically useful for their ability to predict outcomes given a set of patient-specific factors. The larger the dataset or sample size, the more accurate and robust the ML algorithm becomes [222]. These predictive models are key to the advancement of personalized medicine, as the knowledge generated can be used to tailor medical treatment to diverse groups of patients instead of using a “one-size-fits-all” approach based on disease phenotype alone [223]. Given the heterogeneity of individuals with hearing loss, and the variability between human performance and CI technology, the integration of big data and ML into otologic care may provide patients with SSD the most precise treatment strategies, ultimately optimizing post-implantation performance, usage, and perceived benefit.

#### 6.2.1. Current Landscape: What Has Been Done

The past ten years have seen a significant rise in the role of big data and ML in CI research and medical management. However, to date, most research on predictive algorithms has focused on the traditional bilateral CI population, and there is a notable scarcity of models specifically trained and validated on SSD-only datasets [218,219,224]. For example, ML algorithms are increasingly being explored for their potential to accurately guide CI candidacy considerations [225] and post-operative performance expectations based on extensive training with large amounts of pre-operative data collected from previous CI recipients [224]. These include information such as functional MRI data [226], level of hearing loss, auditory test scores, auditory nerve health, and demographics such as age or level of family support [218,227]. These models aim to move beyond traditional regression by capturing complex, non-linear relationships within multi-dimensional datasets, offering a more nuanced prediction of individual outcomes and benefit [227,228].

Furthermore, artificial intelligence (AI) and ML are being applied to optimize aspects of the CI surgery. One example is determining electrode insertion and positioning. Successful CI surgery requires careful navigation of the delicate temporal bone anatomy, which relies on pre-operative CT scans of the head. These images can be used to teach ML algorithms to detect important landmarks and guide the location of the surgery, leading to reduced error rates [229,230]. Additionally, computational models coupled with genetic algorithms, may be used to select the optimal CI electrode array shape, insertion depth, and placement to improve electrical signaling, minimize place-pitch mismatch, and reduce trauma [231,232,233]. ML can also predict post-operative electrode impedances, aiding in fitting sessions to improve CI performance [234].

ML techniques are also being used to develop more sophisticated noise reduction, sound classification, and adaptive processing strategies in the CI program itself [218,235]. Large datasets representing various auditory environments can be used to train neural networks on how to automatically segregate speech from noise: amplifying the desired sound while minimizing the background. When this new algorithm was applied to the CI’s speech processor technology, users demonstrated an increase in speech intelligibility scores as compared to their performance with the processor’s original algorithm [236,237,238]. This holds particular promise for SSD patients, who often struggle with this task due to the asymmetric input. Additionally, ML can be used to automate real-time adjustments of the CI’s sound and signal processing algorithms; its precision is based on both the user’s personal feedback and previous data points [144]. Overall, implementing ML algorithms into CI programming can provide users with a clearer and more comfortable auditory experience, adaptable to their unique listening environments and preferences.

#### 6.2.2. Future Applications and Directions

While most of the research regarding opportunities for ML to improve CI care has been focused on general CI users, all these principles can be extended to enhance treatment of SSD. For instance, a clinician could leverage a predictive model trained on a nationwide SSD database to provide highly personalized pre-operative counseling. By inputting a new patient’s specific variables—such as age, duration of deafness, etiology, and pre-operative audiometric scores—the model could generate a data-driven forecast of that individual’s likely outcome trajectory [228]. Instead of providing general statistics, the clinician could have a more nuanced discussion, such as: “For patients with your specific profile, our model, based on thousands of previous cases, predicts an 85% probability of achieving over 50% word recognition within the first year.” This approach would help set highly realistic expectations, directly addressing a key factor in patient satisfaction and consistent device use [239].

**Figure 3:**
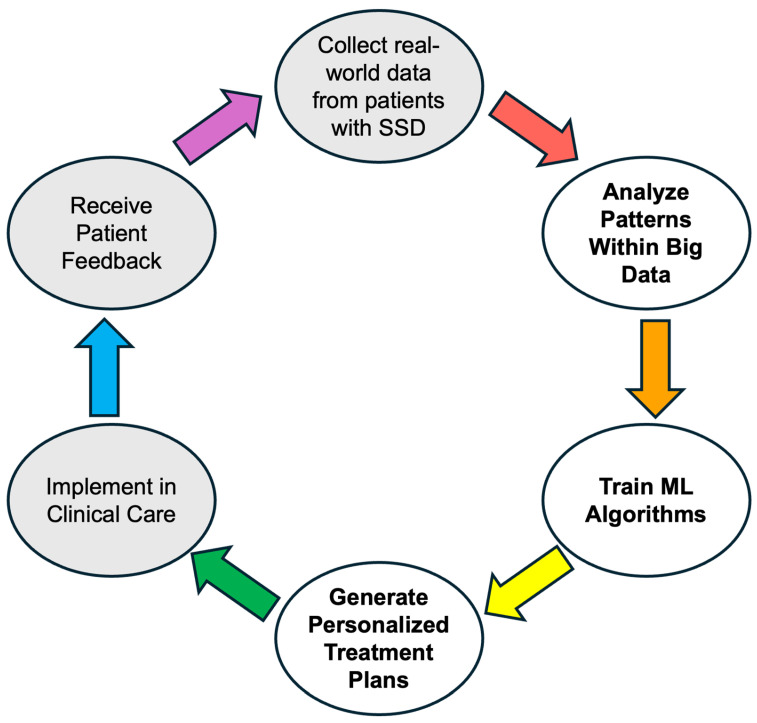
Example workflow of big data and machine learning (ML) applications in optimizing cochlear implant (CI) utility and satisfaction among patients with single-sided deafness (SSD). By organizing and extracting data from large datasets consisting of SSD-only cases, clinicians and their patients can make more accurate and informed predictions regarding treatment strategies, device design, and performance outcomes. Text with gray backgrounds indicate patient-interfacing steps.

Looking ahead, consistent data logging from CI devices used by patients with SSD offers a vast, personalized dataset based on a patient’s own auditory environments and daily usage [131]. This real-world data can be fed into ML algorithms to generate even more precise, personalized recommendations for programming and rehabilitation (Figure 3). AI-driven tools are also emerging for personalized rehabilitation programs, including virtual reality-based training and remote monitoring capabilities, which can adapt to individual user needs and provide tailored feedback [227,240]. Crucially, given the unique etiologies and auditory experiences of SSD patients, it is imperative that future big data and ML initiatives also train algorithms on distinct datasets for SSD and bilateral SNHL. While many advancements have shown potential in the bilateral SNHL field, directly applying these conclusions without considering the profound differences in auditory processing, compensatory behaviors, and overall auditory landscape in SSD would limit the accuracy and clinical utility of personalized guidelines. Creating specialized algorithms based on SSD-specific data will allow for the development of more accurate predictive models and tailored interventions that genuinely address the challenges unique to this distinct patient population.

## 7. Conclusions

Optimizing cochlear implantation in patients with single-sided deafness (SSD) involves a distinct approach that moves beyond the standardized protocols traditionally used for bilaterally deafened individuals. The constant presence of a normal-hearing ear creates a unique set of challenges that contribute to lower device usage and satisfaction. A personalized medicine framework is therefore essential to address these disparities. This approach begins with tailored pre-operative counseling to set realistic expectations and extends to individualized post-operative rehabilitation that moves beyond traditional speech-in-quiet metrics. Incorporating more sensitive assessments, such as music perception—which challenges the auditory system’s ability to process the fine temporal and spectral cues that are poorly transmitted by CIs—can better capture the nuanced auditory processing deficits that drive patient dissatisfaction and guide more effective clinical management. Looking forward, the integration of big data and machine learning offers the potential to further refine this personalized care. By leveraging large-scale clinical registries, predictive models can be developed to forecast individual outcome trajectories, transforming patient counseling and intervention strategies. Ultimately, adopting this tailored, evidence-based approach is critical to overcoming the unique hurdles faced by the SSD population, thereby improving consistent CI usage and enhancing overall quality of life.

## Figures and Tables

**Table 1:** Common etiologies of single-sided deafness (SSD).

Etiology	Definition
Idiopathic SNHL	Cause unknown. May occur suddenly.
Chronic Otitis Media [13]	Persistent or recurrent inflammation and/or infection of the middle ear and mastoid cavity. May cause cholesteatoma: the abnormal growth of keratinizing squamous epithelium in the ear, leading to progressive bone erosion.
Cerebellopontine angle (CPA) tumor [14]	Tumor located at the CPA, the anatomical region at the junction of the cerebellum, pons, and medulla in the posterior fossa (posterior and inferior portion of the brain). Includes vestibular schwannoma, a benign tumor arising from the Schwann cells of CN VIII, which controls both balance and auditory function.
Perilymphatic fistula [15]	An abnormal connection between the fluid-filled inner ear and the air-filled middle ear or mastoid cavity, causing leakage of perilymphatic fluid out of the inner ear.
Cochlear nerve deficiency [16]	Having a cochlear nerve (the auditory branch of CN VIII) either smaller in diameter than normal (hypoplasia) or completely absent (aplasia).
Viral infections	Mumps virus [17]: causes sudden hearing loss by direct viral invasion and subsequent damage to cochlear structures. Congenital cytomegalovirus [18]: transmitted from mother to fetus through the placenta during pregnancy. Causes hearing loss by directly damaging cells and immune-mediated injury to the cochlea and auditory pathway.
Auditory neuropathy spectrum disorder [19]	Hearing loss caused by impaired transmission of sound from the inner ear to the brain, despite intact function of the outer hair cells in the cochlea. Described as “being able to hear but not understand.”

CPA = Cerebellopontine angle. CN VIII = cranial nerve 8, or the vestibulocochlear nerve.

**Table 2:** Cochlear implant (CI) utilization rates and outcomes in patients with single-sided deafness (SSD).

Authors	Study Type	Population (*n*)	Key Findings
Zeitler et al., 2019 [4]	Retrospective cohort study examining pre- and post-op auditory performance and device usage.	Children (9)	(a) Word recognition scores (CNC, MLNT), sentence testing (AzBio, HINT), and bimodal speech reception thresholds all improved after receiving the CI. (b) 8 (88.9%) children were full-time users of their device; the 1 non-user was congenitally deaf but implanted at age 9.5, thus having a long duration of deafness.
Rauch et al., 2019 [38]	Retrospective cohort study comparing CI users with SSD (SSD-CI) to bilaterally deaf individuals using unilateral (Uni-CI) or bilateral CI (Bil-CI)	Children and Adults (206): - SSD-CI (27) - Uni-CI (114) - Bil-CI (65)	(a) SSD-CI of all age groups used their device on average for 8.07 h/day, which was less than both Uni-CI (10.82) and Bil-CI (10.60). Differences between groups were only significant in the working age group (ages 18 to 65). (b) CI use time was similar between age groups within SSD-CI but not within Uni-CI or Bil-CI. (c) The auditory environments in which CIs were activated were overall similar between all groups, but adult SSD-CI used their CIs less than the Uni-CI and Bil-CI groups in quiet environments.
Tavora-Viera et al., 2020 [39]	Retrospective cohort study investigating CI non-use.	Adults (114)	(a) 5 (4.4%) individuals became elective non-users (defined as completely stopping, or refusing re-implantation if needed), with mean time before discontinuation of 11.5 months (range 1.5 to 60 months). (b) Reasons included: distorted sound quality and poor speech understanding through the CI, perceived irrelevance of the CI, unmet expectations of CI benefit, and unwillingness to participate in rehabilitation sessions leading to lack of improvement.
Lindquist et al., 2023 [40]	Retrospective case series examining pre- and post-op auditory performance and CI usage behavior.	Adults (66)	(a) Speech performance scores were significantly higher after CI surgery, peaking at 6 months post-activation. (b) 34 patients (51.5%) had a follow-up appointment with their audiologist after 12 months post-implant. (c) Average daily wear time (mean 8.0 h/day, SD 4.6 h/day) was positively associated with post-operative CNC, AzBio, and CIQoL-10 scores. (d) 9 patients (14%) became non-users or were explanted at last contact. Reasons included: magnet retention issues, lack of perceived benefit in hearing or tinnitus reduction, infection/dehiscence, and magnet pain during MRI scans.
Speck et al., 2021 [41]	Prospective cohort study examining pre- and post-op auditory performance and long-term CI usage behavior	Adults (78): - SSD (41) - AHL (37)	(a) CI improved speech recognition in noise, sound localization, and subjective speech intelligibility and spatial hearing in AHL and SSD. (b) Long-term data (≥5 yrs post-implant) from 76 participants demonstrated CI wear time of 6 to 10 h/day (median: 8 h). (c) 4 (9.8%) individuals in the SSD cohort were elective non-users. Reasons included: CI did not improve speech comprehension, fear of contaminating the device at work, lack of practice with the CI, and lack of subjective benefit.
Muigg et al., 2020 [42]	Prospective cohort study examining hearing-related QoL (HRQoL) measures pre- and up to 2 years post-implantation.	Adults (20)	(a) Cochlear implantation was associated with increased hearing-specific and generic HRQoL among SSD patients within the first 6 months. (b) 2 (10%) individuals discontinued CI use at 12 and 20 months after activation due to perceived poor benefit from lack of adaptation to the device or unmet expectations. Both had long-term SSD (>10 years).
Macielak et al., 2024 [43]	Retrospective cohort study on pre- and post-op audiometric performance and CI usage behavior.	Children (66)	(a) 12 (18%) patients were eventually lost to follow-up. (b) At the last evaluation, only 10 (19%) of the 54 remaining patients were users, 13 (24%) were limited users (>2 but <6 h/day), and 28 (52%) were non-users (≤2 h/day). (c) There was no association between usage and duration of deafness or age of implantation.
Tan et al., 2024 [44]	Retrospective cohort study examining CI usage behavior at 12 and 24 months post-op.	Adults (54): - 12-month follow-up (54) - 24-month follow-up (38)	(a) Mean CI usage was 8.2 h/day (SD 4.2) at 12 months, and 7.0 h/day (SD 5.1) at 24 months post-cochlear implantation. (b) At 12 months, 5 (9.3%) out of 54 individuals were non-users (<1 h/day), while 7 out of 38 (18.4%) were non-users at 24 months—a 9.1% increase in non-use. (c) 15 (27.8%) individuals missed their 24-month follow-up appointment, while 1 was explanted due to worsening tinnitus, not finding the CI useful, and magnet retention issues.
Smith et al., 2024 [50]	Retrospective cohort study examining pre- and post-op auditory performance and CI usage behavior.	Adults (12): - SSD (2) - AHL (10)	(a) Improvements in PTA, CNC word and phoneme, and AZBio scores were significant after implantation. (b) Mean daily CI use was 9.3 h/day (SD 3.3).
Sullivan et al., 2020 [71]	Retrospective cohort study examining pre- and post-op auditory performance and CI usage behavior.	Adults (60)	(a) Improvements in speech understanding, sound localization, and quality of life were found post-implant. (b) 4 subjects became non-users of their CI due to poor device performance after activation. (b) 41.7% (25) of the initial population were lost to follow up by 24 months, 68.3% (41) at 36 months, 76.7% (46) at 48 months, 83.3% (50) at 60 months, and 95% (57) at 72 months post-implant.
Deep et al., 2021 [176]	Retrospective case series examining pre- and post-op auditory performance and CI usage behavior.	Adults (53)	(a) Speech perception in both quiet and noise scores and tinnitus suppression improved significantly after CI. (b) Average daily CI usage (n = 47) was 8.3 h/day (SD 3.5; range 1.5–14). 3 subjects were limited users (<4 h/day) due to difficulty adjusting to the signal. (c) 2 individuals became non-users (reasons included other comorbidities and lack of benefit due to cochlear ossification).
Tan et al., 2022 [177]	Prospective single-arm study comparing pre- and 1-year post-operative auditory performance, and CI usage behavior 4 years post-op.	Adults (8)	(a) PTA in the deaf ear improved significantly after implantation, with post-operative median reaching near-normal levels of 30 dB. (b) 5 (62%) discontinued CI use within 4 years post-implantation. One patient’s reason was the CI worsened his tinnitus, while another found that his tinnitus improved post-surgery even without the CI on and thus perceived no benefit from the CI. Other reasons included follow-up cost concerns and speech through CI sounding distorted.
Ganek et al., 2020 [178]	Retrospective cohort study examining CI datalogging sessions.	Children (23)	(a) Average CI wear time was 6.22 h/day (SD 2.81, range 0.0004 to 14.74). (b) There was no association between wear time and increasing age or hearing experience.
Arras et al., 2022 [179]	Prospective cohort study comparing auditory performance and CI usage behavior of early implanted SSD (SSD + CI) with non-implanted SSD (SSD + NoCI) and NH individuals.	Children (47): - SSD + CI (12) - SSD + NoCI (9) - NH (26)	(a) Prelingual (under the age of 2.5) implantation of children with SSD significantly improves speech understanding in noise and sound localization ability. (b) SSD + CI children wore their speech processor on average 8.9 h/day (SD 2.7, range 2.9 to 12.2). (c) At the end of the study, 8 (75%) of the SSD + CI group were considered regular users (≥8 h/day), 3 were limited users (<8 h/day), and 1 became a non-user due to lack of perceived benefit (though objectively, his scores showed otherwise) and insufficient family support.
Benchetrit et al., 2021 [180]	Systematic review and meta-analysis of 12 studies examining auditory performance and CI usage rates.	Children (119)	(a) Speech perception in noise and quiet and sound localization improved in the majority if children after receiving the CI. (b) 11 studies (101 children) reported device usage metrics: 75 (74.3%) children used their CI regularly, 21 (20.8%) were limited users, and 5 (4.9%) were nonusers. (c) Nonusers had longer duration of deafness and greater average age of implantation compared to both limited and regular users.
Polonenko et al., 2017 [181]	Retrospective cohort study examining CI usage behavior.	Children (7)	(a) Average daily CI use was 7.4 h/day (SD 1.7). (b) Older children had longer average wear times than younger children; a 10% increase in age correlated with a 0.24 h increase in device usage. (c) CIs were used most often in environments that were moderately loud (50–70 dB) or contained speech in noise.
Ramos-Macías et al., 2019 [182]	Prospective cohort study examining pre- and post-op auditory performance and CI usage behavior.	Children (23) with different etiologies: - Congenital SSD (4) - Acquired SSD (19)	(a) CI improved sound lateralization in both groups and speech recognition among the acquired SSD group (as individuals in the congenital SSD group were too young to be tested). (b) Increased binaural benefit from CI was associated with post-lingual deafness and shorter duration of deafness. (c) CI wear time ranged from 6–12 to 10–16 h/day for the congenital and acquired SSD groups, respectively.
Thomas et al., 2017 [183]	Retrospective cohort study examining binaural hearing measures and subjective benefit.	Children (21)	(a) Speech recognition in noise and sound lateralization ability were significantly better in the CI-aided condition compared to unaided. (b) 3 (60%) of 5 subjects with follow-up ≥3 years became limited users or nonusers of CI, reasons being poor hearing benefit or social stigmatization.
Greaver et al., 2017 [184]	Prospective cohort study examining pre- and post-op auditory performance and device usage.	Children (5): - SSD (1) - AHL (4)	(a) Patients were able to achieve speech recognition through their implanted ear even when the better hearing ear was occluded. (b) CI datalogs collected at the most recent follow-up appointment revealed 3 (60%) full-time CI users (≥8 h/day) and 2 (40%) limited CI users (<4 h/day, mostly at school).

AHL = asymmetric hearing loss, NH = normal hearing. CNC = Consonant-Nucleus-Consonant Test, CIQoL = CI Quality of Life test, HINT = Hearing in Noise Test, HRQoL = Hearing-Related Quality of Life test, MLNT = Multisyllabic Lexical Neighborhood Test, PTA = Pure Tone Average.

## Data Availability

No new data were created or analyzed in this study. Data sharing is not applicable to this article.

## Abbreviations

The following abbreviations are used in this manuscript:

CI	cochlear implant.
SSD	single-sided deafness.
SNHL	sensorineural hearing loss.
CPA	cerebellopontine angle.
CN	cranial nerve.
CROS-HA	contralateral routing of signal hearing aid.
BAHA	bone-anchored hearing aid.
PTA	pure tone audiometry.
MRI	magnetic resonance imaging.
CT	computed tomography.
ITD	interaural time difference.
ILD	interaural level difference.
LW	lateral wall.
PM	perimodiolar.
RWI	round window insertion.
DF	default frequency.
ABF	anatomy-based fitting.
CNC	consonant-nucleus-consonant.
CBAT	computer-based auditory training.
CIS	continuous interleaved sampling.
FSP	fine structure processing.
ML	machine learning.
AI	artificial intelligence.
